# Supplementary material for: Emergence of High Pathogenicity Avian Influenza Virus H5N1 Clade 2.3.4.4b in Wild Birds and Poultry in Botswana
Source: Viruses. 2022 Nov 22;14(12):2601. doi: 10.3390/v14122601 (PMC9788244; doi:10.3390/v14122601)
Supplement: Supplementary file 1 [file viruses-14-02601-s001.zip › Table S1.pdf]

SI Table 1. Overview of HPAI surveillance cases submitted to BNVL for post-mortem examination in response to the HPAI state of high alertness and the HPAI outbreaks in Botswana. Samples testing positive are indicated.

Date Reported	BNVL Submission No. 2021/	Species	Location	Status
				Found Dead/in extremis/terminated/ live?
08/04/2021	818	Duck	Selebi-Phikwe	Found Dead- NEGATIVE
08/04/2021	909	Chicken	Tonota Molapo, Palapye	Found Dead- NEGATIVE
09/04/2021	837	Duck	Goodhope	Found Dead- NEGATIVE
22/04/2021	915	Chicken	Ramotswa	Found Dead- NEGATIVE
06/05/2021	1047	Chicken	Tutume East	Found Dead- NEGATIVE
12/05/2021	1106	Guinea Fowl	Rabotsiripa, Gaborone	Found Dead- NEGATIVE
21/05/2021	1171	Chicken	Sebele, Gaborone	Found Dead- NEGATIVE
25/05/2021	1313	Dove	Hukuntsi	Found Dead- NEGATIVE
03/06/2021	1312	Chicken	Bokaa	Found Dead- NEGATIVE
04/06/2021	1338	African Fish Eagle	Habu	Found Dead- POSITIVE
07/06/2021	1336	Wild bird	Ditshwanye, Mochudi	Live- NEGATIVE
09/06/2021	1355	Chicken	Gakuto	Found Dead- NEGATIVE
11/06/2021	1367	Chicken	Mochudi	Found Dead- NEGATIVE
17/06/2021	1423	Chicken; Wild bird	Notwane	Found Dead; Live- NEGATIVE
17/06/2021	1504	Wild Bird	Sehithwa	Live- NEGATIVE
17/06/2021	1511	Chicken	Moshopha, Mahalapye	Found Dead- NEGATIVE
22/06/2021	1548	Chicken	Sekotlo (Mathubudikwane)	Live- NEGATIVE
23/06/2021	1538	Chicken	Kgalapitse, Mochudi	Live- NEGATIVE
24/06/2021	1552	Chicken	Poloka	Live- NEGATIVE
24/06/2021	1553	Chicken	Leshibitse	Live- NEGATIVE
24/06/2021	1568	Chicken	Metsimatale Dam, Kanye	Live- NEGATIVE
24/06/2021	1569	Chicken	Jabula Dam, Kanye	Live- NEGATIVE
25/06/2021	1567	Chicken	Bokaa	Live- NEGATIVE
24/06/2021	1570	Chicken	Maruswa Dam, Kanye	Live- NEGATIVE
24/06/2021	1573	Chicken	Mmamonkge, Kanye	Live- NEGATIVE
25/06/2021	1571	Guinea Fowl	Gasita, Kanye	Found Dead- NEGATIVE
25/06/2021	1572	Chicken	Mosadimogolo, Kanye	Live- NEGATIVE
29/06/2021	1594	Wild Birds	Artesia	Live- NEGATIVE
29/06/2021	1595	Wild Birds	Kaudwane	Live- NEGATIVE
29/06/2021	1663	Chicken	Nokaneng	Found Dead- NEGATIVE
05/07/2021	1652	Chicken	Tshipasetena, Mochudi	Found Dead- NEGATIVE
06/07/2021	1674	Chicken	Shakawe	Found Dead- NEGATIVE
08/07/2021	1697	Wild Bird	NC 26, Maun	Live- NEGATIVE

09/07/2021	1693	Chicken	Sunnyside, Lobatse	Found Dead- NEGATIVE
09/07/2021	1694	Wild Bird	Peleng Dam, Lobatse	Live- NEGATIVE
12/07/2021	1767	Duck	Di Stance, Selebi-Phikwe	Found Dead- NEGATIVE
13/07/2021	1709	Chicken	Gaborone North	Live- NEGATIVE
13/07/2021	1770	Chicken	Mmamanaku, Selebi-Phikwe	Found Dead- NEGATIVE
15/07/2021	1834	Chicken	Palapye	Found Dead- NEGATIVE
26/07/2021	1787	Orpington backyard Chicken	Bokaa	Found Dead -POSITIVE
05/08/2021	1924	Dove; Guinea Fowl	Habu	Found Dead- NEGATIVE
09/08/2021	1975	Wild bird	Lake Ngami, Maun	Live- NEGATIVE
18/08/2021	2096	Chicken	Etsha 13	Found Dead -POSITIVE
18/08/2021	2097	Dove	Sedie	Found Dead -POSITIVE
20/08/2021	2040	Chicken	Bosele ward, Ghanzi	Found Dead- NEGATIVE
26/08/2021	2163	Chicken	Gumare	Found Dead -POSITIVE
27/08/2021	2125	Chicken	Bokaa	Found Dead- NEGATIVE
27/08/2021	2152	Chicken	Mochudi	Found Dead- NEGATIVE
31/08/2021	2145	Chicken	Lobatse	Found Dead- NEGATIVE
31/08/2021	2162	Chicken	Maun	Found Dead- NEGATIVE
01/09/2021	2180	Chicken	Mochudi	Found Dead; Terminated- NEGATIVE
01/09/2021	2181	Chicken	Mochudi	Found Dead; Terminated- NEGATIVE
02/09/2021	2185	Chicken	Artesia	Terminated- NEGATIVE
02/09/2021	2186	Chicken	Mochudi	Found Dead- NEGATIVE
02/09/2021	2187	Chicken	Mochudi	Found Dead; Terminated- NEGATIVE
03/09/2021	2198	Chicken	Phala Camp, Mochudi	Terminated- NEGATIVE
03/09/2021	2199	Wild bird	Kgomodiatshaba	Live- NEGATIVE
03/09/2021	2200	Wild bird	Kgomodiatshaba	Live- NEGATIVE
03/09/2021	2201	Chicken	Gaborone	Found Dead; Live- NEGATIVE
07/09/2021	2228	Chicken	Mmapula, Mochudi	Found Dead; Live- NEGATIVE
07/09/2021	2229	Chicken	Sewelo, Mochudi	Found Dead- NEGATIVE
07/09/2021	2230	Chicken	Pilane	Found Dead- NEGATIVE
07/09/2021	2231	Chicken	Boseja, Mochudi	Found Dead- NEGATIVE
07/09/2021	2249	Chicken	Tsabong	Found Dead- NEGATIVE
08/09/2021	2240	Chicken	Maun	Found Dead- NEGATIVE
08/09/2021	2245	Chicken	Mochudi	Found Dead- NEGATIVE
08/09/2021	2248	Chicken	Shorobe	Found Dead -POSITIVE
09/09/2021	2255	Chicken	Sewelo, Mochudi	Terminated- NEGATIVE
09/09/2021	2231	Chicken	Maun	Found Dead- NEGATIVE
09/09/2021	2332	Chicken	Mohembo	Found Dead- NEGATIVE
10/09/2021	2269	Chicken	Gabane	Found Dead- NEGATIVE
13/09/2021	2289	Chicken	Mochudi	Terminated- NEGATIVE

13/09/2021	2291	Chicken	Mochudi	Terminated- NEGATIVE
13/09/2021	2333	Chicken	Etsha 9	Found Dead- NEGATIVE
13/09/2021	2334	Dove	Etsha 13	Found Dead -POSITIVE
13/09/2021	2348	Chicken	Seronga	Found Dead- NEGATIVE
14/09/2021	2368	Chicken	Gumare	Found Dead- NEGATIVE
14/09/2021	2387	Chicken	Letsepa, Hukuntsi	Found Dead- NEGATIVE
16/09/2021	2355	Chicken	Maun	Found Dead- NEGATIVE
16/09/2021	2366	Chicken	Dalana, Maun	Found Dead- NEGATIVE
16/09/2021	2396	Chicken	Etsha 5	Found Dead- NEGATIVE
17/09/2021	2349	Chicken	Oodi	Found Dead- NEGATIVE
17/09/2021	2350	Chicken	Gaborone	Found Dead- NEGATIVE
17/09/2021	2369	Chicken	Shorobe	Found Dead- NEGATIVE
17/09/2021	2397	Chicken	Etsha 13	Found Dead- NEGATIVE
18/09/2021	2398	Chicken	Gumare	Found Dead- NEGATIVE
18/09/2021	2399	Chicken	Etsha 11	Found Dead- NEGATIVE
18/09/2021	2400	Chicken	Bothatogo, Maun	Found Dead- NEGATIVE
18/09/2021	2401	Chicken	Etsha 6	Found Dead- NEGATIVE
18/09/2021	2402	Chicken	Etsha 13	Found Dead- NEGATIVE
20/09/2021	2365	Dove	Nokaneng	Found Dead- NEGATIVE
21/09/2021	2411	Chicken	Mmashoro	Found Dead; In Extremis- NEGATIVE
21/09/2021	2415	Chicken	Mankgodi	Found Dead- NEGATIVE
24/09/2021	2466	Chicken	Mmantsie	Found Dead- NEGATIVE
24/09/2021	2467	Chicken	Lentsweletau	Found Dead- NEGATIVE
24/09/2021	2469	Chicken	Etsha 5	Found Dead- NEGATIVE
24/09/2021	2470	Chicken	Etsha 5	Found Dead- NEGATIVE
24/09/2021	2471	Chicken	Shorobe	Found Dead- NEGATIVE
24/09/2021	2472	Chicken	Xhaxhao	Found Dead- NEGATIVE
24/09/2021	2476	Chicken	Shorobe	Found Dead- NEGATIVE
27/09/2021	2511	Chicken	Maun	Found Dead- NEGATIVE
27/09/2021	2512	Chicken	Habu	Found Dead- NEGATIVE
28/09/2021	2530	Chicken	Jwaneng	Found Dead- NEGATIVE
29/09/2021	2538	Geese	Mochudi	Found Dead- NEGATIVE
29/09/2021	2539	Duck	Tlokweng	Found Dead- NEGATIVE
