# Supplementary figures and images for: Emergence of High Pathogenicity Avian Influenza Virus H5N1 Clade 2.3.4.4b in Wild Birds and Poultry in Botswana
Source: Viruses. 2022 Nov 22;14(12):2601. doi: 10.3390/v14122601 (PMC9788244; doi:10.3390/v14122601)

Key

Botswana H5N1 June 2021 – September 2021

Eurasian H5N1 2020/2021

African H5N1 December 2020 – December 2021

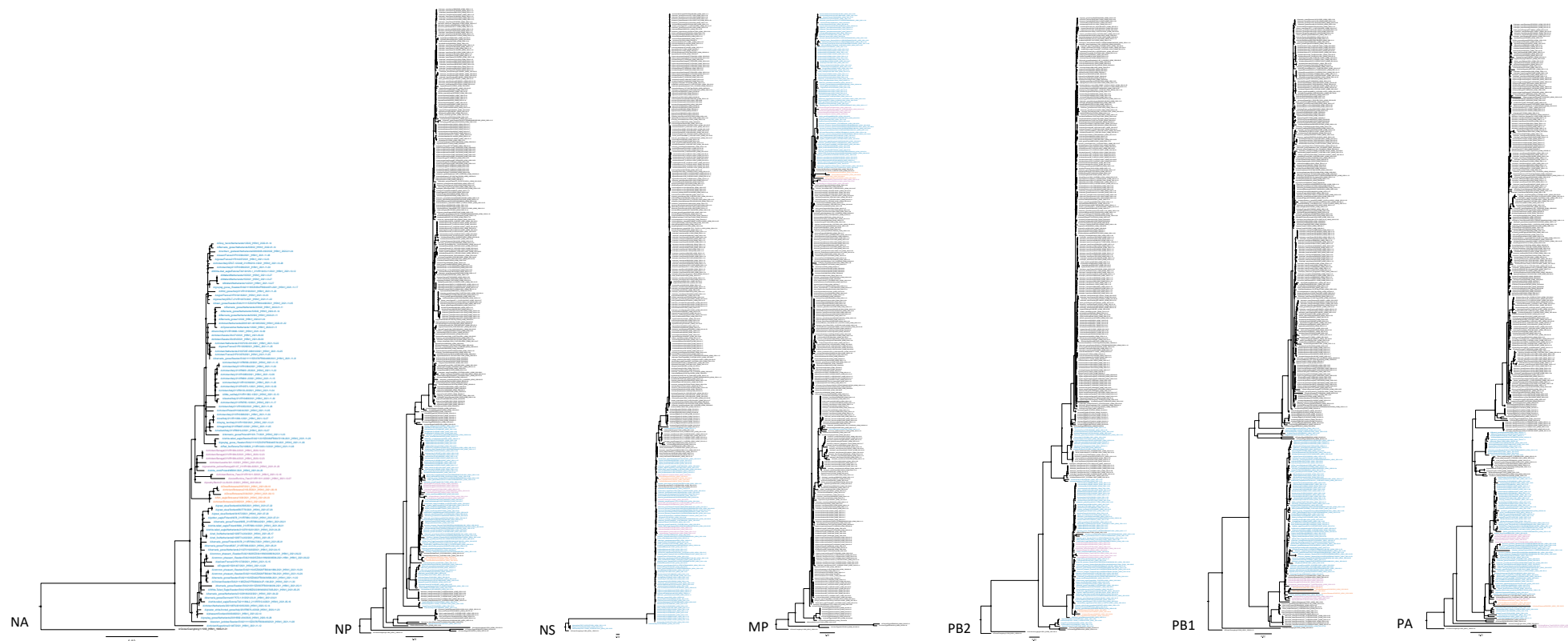

Supplement: Supplementary file 1 [file viruses-14-02601-s001.zip › Figure S1.pdf]
